# Challenges and the Path Forward on Malaria Elimination Intervention: A Systematic Review

**Published:** 2019-06

**Authors:** Khandan SHAHANDEH, Hamid Reza BASSERI

**Affiliations:** 1. Deputy of Research and Technology, Tehran University of Medical Sciences, Tehran, Iran; 2. Department of Medical Entomology and Vector Control, School of Public Health, Tehran University of Medical Sciences, Tehran, Iran

**Keywords:** Systematic review, Malaria elimination, Intervention, Challenges

## Abstract

**Background:**

This systematic review was conducted to highlights key challenges, and outlines important next steps to maximize the potential to contribute to the broader malaria elimination interventions.

**Methods:**

This systematic review on malaria elimination intervention and challenges was undertaken searching six databases, between 1995 and 2018. Inclusion and exclusion criteria were set. The references were collated and categorized according to type of study, intervention, population, and health outcome. Articles selection based on title and abstract, retrieval of full text and additions of articles from reference lists and recommendations from experts. Disagreement in data extraction was solved by consultation of third reviewer.

**Results:**

Overall, 4039 records were examined related to malaria elimination that initially identified by our designated electronic databases search. Overall, 35 studies contained 14 experimental studies (40%) and 21 analytic observational studies (60%) met the inclusion criteria for this review. Studies used a wide variety of malaria elimination interventions. Types of interventions either elimination-focused interventions or general interventions on educational, prevention and treatment of malaria are included. This review pointed out the variety of challenges for eliminate malaria among low and high endemic countries.

**Conclusion:**

Malaria elimination is facilitated by strong health systems, determined leadership, appropriate incentivization, an effective surveillance system, and regional collaborations. We have identified areas for elimination-specific interventions deserve more attention in the conduct and reporting.

## Introduction

Effective malaria elimination interventions are crucial to ensure the elimination of malaria (1). Eliminating malaria is process that requires to understand health behaviors in relation to uptake an intervention, as well as identifying the challenges to implementing effective interventions (1).

Despite tremendous improvement against malaria and reduce mortality rate there is a need to find new ways to reach affected communities with sustained and expanded effective interventions and to ensure they are used appropriately (2–4).

In many countries, malaria elimination program fails, in spite of they have been successful in malaria control programs. As a result of stronger coordination among Role Back Malaria partners, and WHO and increased funding, the majority of endemic countries are on track to meet the malaria-specific Millennium Development Goal target to reduce malaria case incidence by 75%. Accordingly, for a Malaria-Free World, Action and Investment to defeat Malaria 2016–2030 for eliminating the scourge of malaria over the next 15 years and avoiding the resurgence of the disease have been developed and launched (5–7).

Therefore, this systematic review was conducted to provide reliable information in relation to malaria elimination interventions as well as identifying challenges to implementing intervention.

Purpose of review was to provide a status report evidence gaps on good practice and implications for eliminating malaria. These findings can support the efforts towards malaria elimination policy, strategy and practice.

## Methods

### Literature search strategy

Online electronic databases of MEDLINE, CINAHL, PubMed, Web of Science, Global Health and ProQuest Dissertation. These were systematically searched using specified search terms based on the search strategy was developed by the research team, for malaria elimination intervention and challenges to identified studies published in English from Jan 1995 to Apr 2018. The following main Medical Subject Headings [MeSH] were used for the free text search: “Malaria elimination” “elimination challenges” AND “Malaria intervention”. For further specific identifications, studies were restricted to journal articles and targeting “health outcomes” through the additional filter functions of “type of study”, “intervention” and “population”. Finally, we conducted searches of the reference lists of relevant articles to ensure that we did not miss any relevant studies that we had not identified through our selected terms.

### Selection of articles

**Fig. 1:**
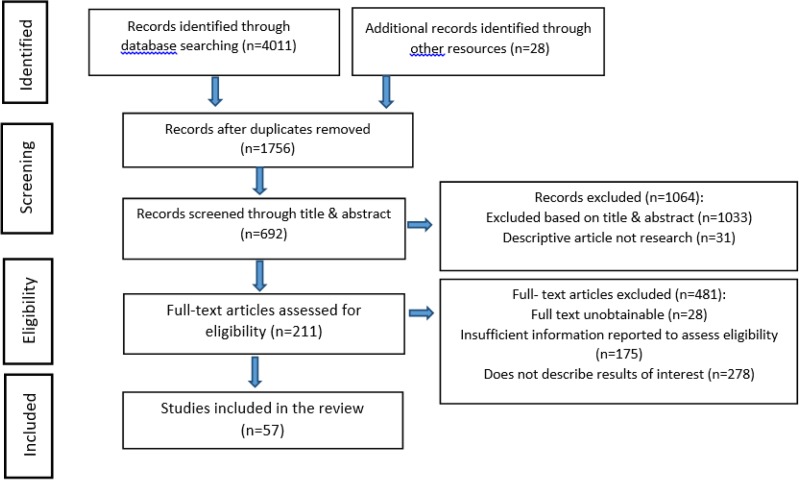
Flow chart of systematic review identification, screening, eligibility, and inclusion

Since a large number of records were identified through the designated search on the online databases and other resources, precise measures were taken for screening and eligibility assessments (Fig. 1).

Studies not published in English were excluded. Including editorials, letters, commentaries, conference abstracts and articles that did not provide sufficient information were not considered a source of information in this systematic review. We excluded all duplicated references. After the duplicates records were removed, selection of articles was undertaken in two phases in this review. To begin with, the title and abstracts of the retrieved literature were reviewed by the two authors to determine the relevant articles, each record was read by two types of research, independently. If the literature met the set inclusion criteria, were included in the study. During this phase, some studies were excluded due to they did not meet the inclusion criteria. Next, the full text of the selected articles in the screening process assessed for eligibility. In eligibility selection phase, the full-text of the articles were retrieved, the studies that passed the eligibility criteria were included and irrelevant studies were excluded due to they do not describe results of interest, insufficient information reported to assess eligibility and full text unobtainable (Fig. 1). We also reviewed references of each article to identify other potentially relevant titles that were then evaluated the same way.

The relevant data from each study were extracted to a standardized form by reviewers. This included data regarding eligible criteria: Studies were eligible if types of studies were: observational studies, experimental studies, and original studies were primary source consist of a plan, program, or screening implemented with the intention to induce behavioral change in a target audience. The populations of interest were: all ages and both sexes particularly, population at risk, children, women and immigrants. Types of outcome measures were: behavior change, policy changes and capacity building were considered.

### Data extraction and synthesis

The data collection process involved abstracting relevant information from the eligible articles and generating summary evidence tables that present the key details and findings for the articles.

Two authors independently reviewed all articles marked for inclusion and extracted data. Using a pre-designed data extraction form, the data were extracted by one reviewer and checked by another. Disagreement in data extraction was solved by consultation of the third reviewer. Then, the articles were grouped by study design, intervention, population and outcome. We employed analytic framework and feedback from the experts. We used a narrative summary structured to examine the evidence.

### Quality appraisal

Different quality assessment tools with quality ratings were used based on the type of study design. The methodological quality of the included studies with particular emphasis on malaria intervention was ranked using Quality Rating forms. For randomized trails, double-blind and description of withdrawals, and for observational studies, selection, comparability and the outcome were assessed. Appropriate research design, appropriate recruitment strategy, response rate reported, sample representative of similar population, objective and reliable measurements used, power calculation and appropriate statistical analysis were looked into when evaluating the quality of cross-sectional studies. National CASP Appraisal Tool was also used as assessment tool for qualitative study which examined rigor, credibility, and relevance. The quality assessment was carried out independently by two reviewers and consultation of third author in the event of disagreement.

## Results

Of the 4039 records initially identified by our designated electronic databases search and after excluding for duplications, 1756 records were identified. Overall, 1064 records were excluded based on title and abstract, of the 692 records screened. Of the 211 full-text articles assessed for eligibility, 481 articles were excluded. Thirty-five articles were included after reading the full text, including experimental studies and analytic observational studies met the inclusion criteria for this review (Fig. 1). Overall our sample contained 14 experimental studies (40%) and 21 analytic observational studies (60%) (Table 1).

**Table 1:** Summary of included studies

***Ref. ID***	***Author, Year***	***Type of study***	***Intervention***	***Population***	***Outcome***
1	Wirth et al. 2018	Observational	Identifies gaps in skills & resources	Malaria programme personnel	Expanded training for malaria elimination
**2**	Hlongwana et al. 2018	Observational	Personnel’s experiences to implementing strategies	Malaria programme personnel	Feasibility of malaria elimination goal
**3**	Sahan et al. 2017	Observational	Treatment, Mass Drug Administration	Rural & healthcare staff	Community engagement. Improve health
**4**	Nguyen T-N 2017	Experimental	Mass Drug Administration	Rural	Increase participation
**5**	Zhu et al. 2017	Experimental	Combination intervention of LLINs & outdoor ATSB	Rural	Reduce transmission
**6**	Yalew et al. 2017	observational	Rapid Diagnostic Tests(RDTs) & serology assays	Rural & Urban	Reduce transmission
**7**	Wotodjo et al. 2017	observational	Artemisinin-based Combination Therapy and long-lasting insecticide-treated nets.	Rural & Urban	Programme targeted migrant workers
**8**	Win et al. 2017	observational	Assess the migration patterns, malaria treatment-seeking preferences	Migrant workers	National Malaria Control Programme targeted migrant workers
**9**	Zhang et al. 2017	observational	Update surveillance tools in malaria transmission	Rural & Urban	Development of appropriate surveillance strategies and WHO certification
**10**	Zhang et al. 2016	observational	Assess the malaria epidemic risk in China’s land border regions	Rural & Urban	To achieve and maintain malaria elimination
**11**	Zhou et al. 2016	observational	Update surveillance tools	Residents	Indigenous transmission elimination
**12**	Larsen et al. 2015	Experimental	Testing and Treatment with Rapid Diagnostic Tests & Artemether-Lumefantrine	Rural & Urban	Reduce transmission
**13**	Shahandeh et al. 2015	Observational	Assess community participation	Native and immigrant groups	Community involvement and capacity building
**14**	Shahandeh et al. 2015	Observational	Perceptions of health workers in relation to eliminating malaria	health workers	Practice to health providers
**15**	Cook et al. 2015	Experimental	Mass screening and treatment RDTs	Residents hot spot	Reduce transmission
**16**	Parham&. Hughes 2015	Experimental	Develop a model for assessing Cost-effectiveness of malaria interventions	Residents	Cost-effectiveness of malaria interventions, IRS & LLINs
**17**	Shahandeh et al. 2014	Observational	Cultural model for practices malaria prevention	Resident and immigrants	Cultural effect on prevention and treatment of malaria
**18**	Silal et al. 2014	Experimental	Scale-up of vector control, mass drug administration, mass screen & treat campaign	Residents	Applied model to reduction local and foreign reservoir infections,
**19**	He et al. 2014	Experimental	Behavioral change communication	Mountain worker	improved the ability of mountain workers to avoid malarial infection
**20**	Pindolia et al. 2014	Observational	Endemic regions model cross-border migration & movement	Immigrant	Facilitate intervention & collaborative policy decisions
**21**	Akoria & Arhurdese. 2014	Observational	Scale-up of case management, mass screen & treat campaign	Residents of Urban area	Improve malaria therapy, elimination strategies
**22**	Adhikari S. R. 2014	Observational	Improvement in Health system function	Residents	Protection people from poverty induced by health care costs
**23**	Cancer et al. 2013	Experimental	Diagnostic tools asymptomatic carriers	Rural residents	Switch from passive to active malaria case detection
**24**	Tobgay et al. 2013	Experimental	Community-directed interventions	Residents	Improving knowledge, attitude and practice
**25**	Basseri et al. 2012	Observational	Identify community sleeping and vector blood feeding behavior	Residents	Promoting community self-protection against mosquito bites
**26**	Yangzom et al. 2012	Observational	Prevention and surveillance	at-risk Bhutanese and migrant workers	Prevent importation of malaria
**27**	da Silva-Nunes et al. 2012	Observational	Malaria intervention policies in Brazil & Peru	Rural residents	sustainable malaria interventions
**28**	Kobylinski et al. 2011	Experimental	Scale up to elimination program Ivermectin MDA	Rural residents	Parasite transmission control
**29**	Tynan et al. 2011	Observational	Treatment-seeking behaviour	Rural residents	Early diagnosis & prompt Effective case management
**30**	Shahandeh et al. 2010	Observational	Protection & mosquito biting surveillance	Rural residents	Self-protection communities & health workers
**31**	Basseri et al. 2010	Observational	Comparing malaria transmission and protection behaviour	Residents & foreign immigrants	Difference in lifestyle & protective behavior of two community effect on prevalence of malaria.
**32**	Atkinson et al. 2009	Observational	Coverage and usage of LLIN in communities	Head of households, Primary caregivers, Youths	acceptability and preference LLIN
**33**	Bhattarai et al. 2007	Observational	ACT and ITNs interventions	Rural children	Reduction in transmission
**34**	von Seidlein L 2003	Experimental	Mass Drug Administration	Rural residents	Reduction transmission
**35**	Rojas et al. 2001	Experimental	Planned, implemented & evaluated	Households in coastal areas	Evaluate Malaria Control Program, Strengthen diagnosis & treatment network

Studies used a wide variety of malaria elimination interventions. Types of interventions were either elimination-focused interventions involved scale-up of vector control (8–17), mass drug administration (9, 18–23), mass screen and treat campaign (9, 21, 23–25), and foreign source reduction and malaria movement (9, 12, 24, 26–32), or general interventions on educational, prevention and treatment of malaria. For example, training private health care providers (33), using behavior change communication prevention malaria strategy (22, 29, 32–39) and empowering community on malaria by community -directed educational intervention (30, 33, 34, 40, 41), diagnostic tools for identifying very low parasite densities in asymptomatic carriers (24, 25).

Malaria transmission as a controversial subject was investigated in majority of studies. The analysis of observational studies on malaria elimination help understanding community barriers to participating in malaria elimination interventions such as mass drug administration trials is vitally important (22). The majority of the reviewed studied (34 studies) provided reliable information in relation to develop new intervention strategies for malaria elimination.

The study locations are mostly in low and middle-income countries. More than half of the studies were undertaken in the Asia Pacific region (48.5/35=51%). Of the remaining studies, 15 were undertaken in the Africa region (15/35=42%), and 3 in Amazonian countries (3/35=8.5%). Table 2 shows the characteristics of the studies locations in which the reviewed studies were conducted in region and countries.

**Table 2:** The number of studies conducted on challenges of malaria elimination intervention based on location

***Region***	***Countries***	***Number of studies***
Africa	Zambia, Kenya, Tanzania, Mpumalanga, Senegal, Uganda, Gambia, Nigeria, Ethiopia	No.=15
Asia/Asia pacific	Vietnam, Myanmar, China, Cambodia, Bhutan, Nepal, Solomon Islands, Iran	No.=17
Amazonian countries	Colombia, Brazil, Peru, Vanuatu	No.=3

Research-based evidence confirmed the variety of challenges in malaria elimination with regards to malaria transmission coverage. A major challenge for elimination was identified in a number of studies (9, 21, 24, 25, 28, 29, 42) were prevention of imported cases due to human reservoir of malaria parasites. Moreover, asymptomatic infections were highlighted as another main challenge (21, 24, 32). We also clustered the reviewed studies based on local, national and international levels. Nineteen of studies were conducted at local level (54%) and 11 studies at national level (32%). Only 5 studies were conducted at international level (14%). Levels of intervention in malaria elimination will be difference and complexity, so it would be better to consider in the context of elimination setting to develop effective malaria elimination interventions.

Another finding in our review was the number of study in terms of community defined. Community defined fell into three categories. These categories included geographical location (23/35=66%), social ties which refer to family and support groups (6/35=17%) and social diversity which refer to race and ethical origin (6/35=17%).

For many countries that implement malaria elimination program, finding an effective strategy for managing the problem was a major challenge.

Elimination of malaria has been identified as regional priority program. Eliminating malaria for African countries requires to enable the population at risk to have equal access to free malaria treatment-related drugs. While among other countries in Asia and Latin America, their approach to the problem involves advocate for effective malaria interventions such as prevention and management of imported malaria infections. Data concerning malaria transmission including malaria infection reservoir were presented in 14 articles. Seven articles were recorded from Africa (9, 21, 24, 28, 43, 44), with three articles from Latin America (11, 42) and four articles from Asia (29). However, in general, these publications were primarily aimed at reducing the human reservoir of malaria parasites as they identified a major challenge for malaria elimination.

We grouped the outcome measures of reviewed articles according to the learning, behavior change to decreases in local and imported infections and improved health status. Several studies measured outcomes learning of target population. These outcomes related to building awareness of malaria elimination of population at risk (29), general public, ex-patients, migrant population and healthcare workers. As a result, involvement of community in malaria elimination efforts increased.

Moving toward elimination of malaria involves understanding of human and mosquito behaviors. Behavior change in human and mosquito was measured in nine of the studies. These measures included general measures of adherence to behavioral change as well as care management behaviors such as the use of health services for diagnosis and treatment, taking antimalarial drugs, use of mosquito bet net and understanding of mosquito behaviors was considered for resistance to insecticides, and find out which mosquitoes are most susceptible to indoor spraying (8, 20, 21, 24, 29, 33).

Additional challenge confronting the malaria elimination is how to raising awareness and encourage communities/stakeholders to engage in interventions. There is an emerging consensus that a range of promotional communications can positively change knowledge, attitudes, and behaviors of community members on malaria elimination.

## Discussion

The results presented that there are numerous challenges that must be overcome to achieve effective malaria elimination interventions.

Our findings highlight the most common rational for elimination-focused interventions was accurate diagnosis and prompt effective treatment of malaria in high endemic area demonstrated over years. Although many studies reporting mass screening diagnosis and treatment uncomplicated malaria was the first intervention trialed against malaria, several interventions recently received increasing attention such as vector-based interventions, foreign source reduction, and community-based intervention for malaria elimination.

Some interventional and observational studies were included here, by presenting the successes and failures of their elimination efforts provide an opportunity for other countries to learn from their experiences with elimination. Research-based and practice-based evidence confirmed the challenges of preventing and management of imported malaria infections malaria transmission for countries that share lengthy land borders with neighboring endemic countries. In these countries elimination face the challenge of continued malaria importation were attributed to a variety of causes, including the movement of humans and mosquitoes and the parasites they carry, for example in East Africa, ‘Hotspots’ of origin-specific immigrants from neighbouring countries were identified for Kenya, Tanzania and Uganda with different migration patterns (28). In Bhutan, for example, plans to implement screening at border points to prevent importation of malaria and to targeted prevention and surveillance efforts towards at-risk Bhutanese and migrant workers in construction sites (27). This was confirmed by a recent study (45) which suggested the need for more studies to prevent and management of imported malaria infections from neighbouring countries. Effective solutions will need to address challenges due to facilitate intervention planning, resource allocation and collaborative policy decisions for cross-border movements and migrations.

In order to accurately monitor progress towards the elimination should strengthen systematic operational research thorough understanding of transmission dynamics and behavior factors associated with social acceptability to investment in effective interventions.

These results are in agreement with findings in earlier systematic reviews (46, 47). As in recent systematic reviews on adherence to antimalarial drugs were found behaviors associated with patient adherence to antimalarials and patterns of antimalarial drugs for the treatment of malaria were based on the local malaria situation. For example in African countries, Artemisinin-based combination (47), were appropriate therapy, while in Latin American and Caribbean populations implication anti-malarial Primaquine (46), for malaria elimination has proven to be effective.

There was an increase in the proportion of articles relating to potential changes that may affect elimination efforts. Evidence confirmed the diversity of approaches was employed to eliminate malaria. Successful interventions are usually based on an integration of various approaches through involving many stakeholders and implementing in diverse setting.

Suppressing the malaria vector is a necessary element of any malaria elimination program. It considers interrupt transmission methods (11, 25) and discusses insecticide resistance (14). However, addressed challenges such as vector resistance appear historically to have been of secondary importance for elimination to infection reservoir.

The strengths of study are that the majority of evidence represents the approaches and challenges toward eliminating malaria according to local conditions. In addition, the prevalence of malaria varied greatly across reviewed studies included those were conducted in countries with a high malaria burden and large populations, or in countries with low and moderate endemicity.

This review is subject to several limitations, an examination of which may inform the design and conduct of future studies on malaria elimination. First, the 35 studies identified were widely heterogeneous. Among the reviewed studies, the type of study design and intervention setting varied. Second, malaria interventions deal with relatively complex causal pathways, as did the use of malaria elimination strategies. Lastly, among all the vector-borne diseases, only malaria was selected for the review. As a result, we may have eliminated studies that have well-designed controlled trials.

## Conclusion

This review highlights the existing evidence based on challenges to malaria elimination intervention by identify research priorities as fundamental parts of developing guideline for malaria elimination. This information also can guide health planners and decision-makers in choosing the most appropriate combination of curative and preventive measures to eliminate malaria with the aim of enhancing the partnership between researchers and decision-makers.

Further studies are required in order to make informed policy choices and to improve the delivery of effective malaria intervention.

## Ethical considerations

Ethical issues (Including plagiarism, informed consent, misconduct, data fabrication and/or falsification, double publication and/or submission, redundancy, etc.) have been completely observed by the authors.
